# Ultrasonographic Prevalence and Factors Predicting Left Ventricular Diastolic Dysfunction in Patients with Liver Cirrhosis: Is There a Correlation between the Grade of Diastolic Dysfunction and the Grade of Liver Disease?

**DOI:** 10.1100/2012/615057

**Published:** 2012-07-25

**Authors:** Vasilios Papastergiou, Lamprini Skorda, Phillipos Lisgos, Nikolaos Papakonstantinou, Tsampikos Giakoumakis, Konstantinos Ntousikos, Stylianos Karatapanis

**Affiliations:** ^1^Liver Clinic, First Department of Internal Medicine, General Hospital of Rhodes, 85100 Rhodes, Greece; ^2^Department of Internal Medicine, “Konstantopoulio” General Hospital, 14233 Athens, Greece; ^3^Cardiology Department, General Hospital of Rhodes, 85100 Rhodes, Greece

## Abstract

Presence of cardiac dysfunction has been associated with an unfavorable prognosis in patients with liver cirrhosis. In the present study, 92 consecutive, newly-diagnosed patients with liver cirrhosis were prospectively evaluated. Liver disease was graded according to the modified Child-Turcotte-Pugh (CTP) score whereas left ventricular diastolic function was assessed by Doppler-echocardiography and graded (Stage 0 to 4) according to current guidelines. Overall, DD was diagnosed in 55/92 (59.8%) patients [DD-stage-1: 36/92 (39.1%), DD-stage-2: 19/92 (20.6%)]. Prevalence of DD-stage-1 among the different stages of liver cirrhosis was: CTP-class A: 11/29 (37.9%), B: 15/39 (38.5%), C: 10/24 (41.6%), (*P* > 0.05 in all comparisons), whereas for DD-stage-2 the corresponding proportions were CTP-class A: 3/29 (10.3%), B: 5/39 (12.8%), C: 11/24 (45.8%), (*P* = 0.0009 between CTP-class C versus A and B). Age > 53 years (Odd's Ratio [OR]: 4.2; 95% confidence interval [CI]: 1.5–12.1) and CTP-class C (OR: 4.6; 95% CI: 1.1–20) could independently predict DD. No relation between presence of DD and the etiology of the liver disease was found. We conclude that DD is a common feature in liver cirrhosis. DD-stage-1 is fairly prevalent among all CTP-classes whereas DD-stage-2 seems to be characteristic of the advanced liver disease (CTP-class C). A high level of awareness for the presence of the syndrome is required, especially if cirrhotic patients are CTP-class C and/or of older age.

## 1. Introduction

Hemodynamic alterations characterized by increased cardiac output and decreased systemic vascular resistances are well known to be extremely common among patients with liver cirrhosis [1, 2]. During the past 2 decades, various observations have indicated the presence of a latent cardiac dysfunction which includes a combination of blunted myocardial contractility, altered diastolic relaxation, and electrophysiological abnormalities, all occurring in the absence of a concomitant heart disease [3–5]. This clinical entity, called “cirrhotic cardiomyopathy,” has been repeatedly shown to have a negative prognostic impact, especially on the outcome of invasive procedures such as surgery, transjugular intrahepatic portosystemic shunt insertion (TIPS), and liver transplantation [6–9]. Diastolic dysfunction (DD), caused by decreased left ventricular compliance and relaxation, is believed by some authors to be present virtually in every patient with cirrhotic cardiomyopathy [10–13]. Doppler echocardiography directed to detect simple echocardiographic indices, such as a reduced early/late filling velocity ratio (E/A ratio ≤1), has been proposed as an available screening test for the diagnosis of the syndrome. However, according to current recommendations, DD can be echocardiographically graded in four stages [14], and to date the prevalence of the different stages of DD among patients with cirrhosis remains unknown. Although sparse data suggests an increasing prevalence of DD in the advanced stages of liver disease (decompensated cirrhosis with large ascites), a clear correlation between stage of liver cirrhosis and stage of cardiac dysfunction has not yet been established. Notably, a reversion of the E/A ratio (normally >1) has been widely used as the only definition criterion for the diagnosis of DD in most of the previous studies evaluating cardiac function in patients with cirrhosis. It is the opinion of the authors that pseudonormalization of the E/A ratio which occurs in the advanced stages of DD (Stage 2 or more) could probably represent a source of confounded results creating bias in the estimation of the prevalence rates of that entity. Therefore, more studies assessing prevalence of cardiac dysfunction in cirrhosis while accurately staging the heart disease and stabilizing its prognostic role for the clinical outcome of the cirrhotic patient are required.

The present study aimed to assess the echocardiographic prevalence of DD among a population of cirrhotic patients and to investigate whether a correlation between stage of cardiac dysfunction and stage of liver disease could be established. Furthermore, univariate and multivariate regression analyses were performed to identify independent predictors for the presence of DD. To our knowledge, this is the first paper regarding features of cirrhotic cardiomyopathy in our country.

## 2. Patients and Methods

Consecutive newly diagnosed patients with cirrhosis of the liver, presenting to the liver clinic of our centre, between July 2007 and March 2010, were included in this prospective study. Diagnosis of cirrhosis was based on clinical examination, abdominal ultrasonography, and laboratory findings. After excluding patients with recent variceal bleeding, history of heart disease (ischemic, congenital, rheumatic), hypertension, cardiomyopathy, thyroid disorders, or baseline ECG abnormalities (e.g., Left bundle branch block, left ventricular hypertrophy, preexcitation syndromes), a total of 92 cirrhotic patients were eligible for the study. Patients with an alcohol etiology were included in the study after at least six months of abstinence. A full medical history including details and duration of the etiologic factor of the liver disease was obtained from all subjects whereas presence or absence of jaundice, ascites, splenomegaly, and hepatic encephalopathy was noted. Hemoglobin, platelet count, prothrombin time, and liver function tests including serum bilirubin, albumin, and transaminases were estimated. Patients were assigned to 3 prognostic groups according to the modified Child-Turcotte-Pugh (CTP) score (CTP class A, CTP class B, and CTP class C).

All subjects underwent echocardiography (M-mode, 2D, and Doppler study) to assess cardiac structure and function. The following measurements were obtained: (1) left ventricular performance parameters: left ventricular internal dimensions in systole (LVESD) and diastole (LVEDD). Ejection fraction (EF) and the percentage of fraction shortening of internal diameter (% SF) were calculated. (2) Doppler measurements: peak early (E) and late (A) ventricular filling velocities and E wave deceleration time (EDT) were measured, and the early to late filling velocity ratio (E/A ratio) was calculated. Isovolumic relaxation time (IVRT), measured by simultaneous Doppler and M-mode echocardiography, and left atrial diameter were also estimated. The cardiologist T. Giakoumakis who performed and interpreted the echocardiograms was blinded to the clinical and laboratory status of the patients. Left ventricular diastolic dysfunction was sonographically diagnosed according to current recommendations [14, 15] and was graded as follows.

**Stage 0 (Normal)**

E/A ratio between 1 and 2, EDT between 150 and 240 ms, IVRT between 70 and 90 ms, and normal left atrial diameter (<4.1 cm in men and <3.9 cm in women).

**Stage 1 (Impaired Relaxation)**

E/A ratio < 1.

**Stage 2 (Pseudonormal)**

E/A ratio between 1 and 1.5, IVRT > 90 ms and mild to moderate left atrial enlargement (4.1–5.1 cm in men and 3.9–4.6 cm in women).

**Stages 3-4 (Restrictive)**

E/A ratio > 1.5 and at least two of the following: (a) EDT < 150 ms, (b) IVRT < 70, and (c) severe left atrial enlargement (≥5.2 cm in men and ≥4.7 cm in women).

Written informed consent was obtained from all participants. The study protocol was approved by the local ethics committee and conforms to the Helsinki declaration.

### 2.1. Statistical Analysis

Collected data was analyzed using the SPSS version 19 for windows statistical package. Noncategorical values are given as mean ± standard deviation or as median and range. Fischer's exact test and Student *t*-test were used as appropriate to compare between groups. *P* values lower than 0.05 were considered statistically significant. Univariate analyses were done using the Mann-Whitney *U* test for continuous variables and the Chi-square test for categorical variables. All variables found significant on univariate analysis were included as candidate variables in a multivariate regression analysis model to identify independent predictors for the presence of left ventricular diastolic dysfunction.

## 3. Results

Ninety-two eligible patients (62 males; mean age 53.2 ± 11.3 years) with cirrhosis of the liver were included in the study. The etiology included hepatitis B virus (32 patients; 34.8%), followed by hepatitis C virus (27 patients; 29.3%), alcohol (19 patients; 20.6%), autoimmune hepatitis (5 patients; 5.4%) whereas the cause remained unknown in 9 patients (9.8%). There were 29 patients (31.2%) in CTP class A, 39 patients (41.9%) in CTP class B, and 24 patients (25.8%) in CTP class C.

Echocardiographic findings of left cardiac chamber diameters and systolic performance indices with regard to the different grades of liver disease are shown in Table 1. Left ventricular internal diameters and left atrial sizes were comparable among the three prognostic subgroups of cirrhosis (mean ± SD LVESD, LVEDD, and LAD: 3.2 ± 0.2 mm, 5 ± 0.1 mm, and 3.3 ± 0.3 mm, resp.). Overall, mild to moderate left atrial enlargement was found in 34 out of 92 (36.9%) patients participating in the study. The measured parameters regarding systolic performance of the left ventricle were comparable among the three CTP prognostic subgroups, even though there was a trend for lower EF and % SF rates with the advancement of liver disease (Table 1). The comparative assessment of Doppler-derived diastolic filling indices is shown in Table 2. There was a significant increase in A-velocity in the CTP class B and CTP class C subgroups as compared to CTP class A (*P* < 0.001 and *P* = 0.001, resp.), whereas EDT was significantly increased in CTP class C patients versus CTP class A (*P* < 0.0001) and CTP class B (*P* = 0.0001). 

Overall, diastolic dysfunction was diagnosed in 55/92 (59.8%) of the patients studied. Diastolic dysfunction grade 1 was found in 36/92 (39.1%) and DD grade 2 in 19/92 (20.6%), whereas no cases of DD grade 3 or DD grade 4 were identified. Occurrence of DD among the different stages of liver cirrhosis is shown in Table 3. Prevalence of any stage of DD was 14/29 (48.3%) for CTP-class A, 20/39 (51.3%) for CTP-class B, and 21/29 (87.5%) for CTP-class C (*P* = 0.001 between CTP-class C versus A and B). Prevalence of DD Stage 1 was CTP class C > class B > class A, although statistical significance was not reached. On the contrary, prevalence of DD Stage 2 was significantly higher among patients classified as CTP class C versus CTP class A and B (*P* = 0.0009). Mean CTP score ±SD for patients with DD Stage 2 was 10.2 ± 2.1, significantly higher than that for patients diagnosed with DD Stage 1 (7.8 ± 1.8; *P* < 0.0001) and for patients without DD (7.1 ± 5.4, *P* = 0.02).

On univariate analysis, age > 53 years (i.e.; greater than the mean of the population studied), presence of ascites, CTP class (C versus A/B), and prophylactic treatment with betablockers were significantly associated with the presence of any stage of diastolic dysfunction (Table 4). On multivariate regression analysis, age > 53 years (Odd's Ratio [OR], 4.2; 95% confidence interval [CI], 1.5–12.1) and CTP class C (OR 4.6; 95% CI, 1.1–20) were found to be the only independent predictors for the presence of left ventricular diastolic dysfunction (Table 5).

## 4. Discussion

Many aspects of cardiac structure and function have been described to be abnormal in cirrhosis including histological and structural alterations, systolic and diastolic dysfunction, and electrophysiological changes [16, 17]. Among this wide spectrum of abnormalities, diastolic dysfunction in particular has been shown to provide important information regarding prognosis, especially when procedures implicating significant volume changes, such as TIPS insertion, should be performed [7, 8]. At the same time, DD is known to be associated with an aggravation in the clinical course, probably by playing a role in the pathogenesis of sodium and fluid retention [16] and therefore leading to a slower mobilization of ascites. During the last 25 years, there have been numerous studies pointing out the importance of cardiac dysfunction in the prognosis of patients undergoing liver transplantation [9, 18, 19]. In support of the hypothesis of a direct relationship between liver disease and myocardial function is the discovery that diastolic dysfunction can be completely reversed after liver transplantation [20].

Our study shows that some grade of left ventricular diastolic dysfunction is common among patients with cirrhosis and can be detected by Doppler echocardiography in almost 60% of cases. This rate is comparable to that found by a recent study using tissue Doppler imaging [21]. Abnormal left ventricular diastolic filling was graded as Stage 1 (characterized by an impaired diastolic relaxation pattern) in almost two-thirds of the patients studied (36/55; 65.5%) whereas the rest of the cases were graded as Stage 2 (characterized by the more severe pseudonormal filling pattern). A complex pattern of diastolic dysfunction in cirrhosis which was most pronounced in the advanced stages of liver disease (decompensated patients with tense ascites) has been previously reported by other authors [10, 11, 20], and, indeed, our finding of CTP class C as an independent predictor for the presence of any grade of DD largely supports these older observations. However, it is worthy of note that we could not detect any significant difference in the prevalence of DD Stage 1 among the three prognostic subgroups, in contrast with DD Stage 2 which has been shown to be clearly associated with CTP class C (Table 3). Accordingly, a higher mean CTP score was found for the patients diagnosed with a Stage 2 DD (10.2 ± 2.1) as compared to those diagnosed with DD Stage 1 (7.8 ± 1.8, *P* < 0.0001) and those without DD (7.1 ± 5.4, *P* = 0.02). Moreover, in the present study, as in older reports [7, 21], no relationship between the presence of diastolic dysfunction and the etiology of liver disease was found.

Echocardiographic evidence of mild to moderate left atrium enlargement was found for 36/92 (36.9%) of the studied population. Importantly, a correlation between left atrial size and intrapulmonary right to left shunt, characterizing hepatopulmonary syndrome [22], was observed in a previous report [23]. Although a detailed evaluation of left ventricular geometry was out of the scope of the present study, it is worthy of note that mean left ventricular diameters were normal and comparable among the different prognostic subgroups of patients with cirrhosis (Table 1). Multiple factors have been involved in the development of the increased ventricular stiffness seen in DD, including a combination of myocardial hypertrophy, fibrosis, and subendothelial oedema [3, 16, 17]. However, overt structural changes in the left ventricle are not a prerequisite for diastolic dysfunction. Indeed, in older studies with a smaller number of patients, normal left ventricular morphology in patients with cirrhosis and cardiac dysfunction was observed [24–26]. Interestingly alternative pathogenetic mechanisms such as a decrease in cardiomyocyte metabolism have been recently proposed in order to explain diastolic dysfunction and its reversibility after liver transplantation [27].

Parameters regarding left ventricular systolic performance were normal in all studied patients. In contrast with diastolic dysfunction which is common in patients with cirrhosis and can be revealed by echographic studies performed at rest, prevalence of systolic dysfunction appears to be variable and usually needs stress to elicit it [27]. Left ventricular EF has been reported to be normal in some studies [24, 28–30], increased in others [13, 31, 32] and decreased only in one study of patients with cirrhosis and ascites [11]. A relative increase in the EF of patients with cirrhosis could be explained by the hyperdynamic circulation as a result of splanchnic vasodilatation. However there was not a group of healthy controls included in our study, and therefore we could not perform a comparison in order to detect that phenomenon.

According to the results of multivariate regression analysis performed in our study, older age (>53 years), other than CTP class C disease severity, was found to be an independent predictor for the presence of DD. It is well known that prevalence of DD varies directly with the mean age of the population [33]. Patients with altered diastolic function are likely less able to use the Frank-Starling mechanism, and therefore it would seem logical to conclude that procedures implicating rapid volume changes or an increase in left ventricle afterload should be managed with particular caution in cirrhotic patients of older age. 

There are several limitations for which our study may be criticized. A major drawback is that age correction of the E/A ratio was not performed, whereas more sophisticated echocardiographic tools (e.g., tissue Doppler imaging), which could have increased the reliability of our results, were not used. Hence, the probability of type 1 error could not be excluded, but, on the other hand, we believe that our results represent useful data extracted in a real clinical practice setting. Further studies are therefore required to better assess the prevalence and the characteristics of left ventricular dysfunction in cirrhosis, as well as the prognostic impact of diastolic dysfunction in the clinical outcome of the patient with liver disease.

In conclusion, diastolic dysfunction is quite common among patients with liver cirrhosis, occurring in almost 60% of this population. DD Stage 1 is fairly prevalent among all stages of liver disease whereas DD Stage 2 seems to be characteristic of the more advanced stages of liver disease (CTP class C). A high level of awareness for the presence of the syndrome is required, especially if cirrhotic patients are CTP class C and/or of older age.

## Figures and Tables

**Table 1:** Comparative assessment of echocardiographic measurements and left ventricular systolic performance parameters with regard to severity of liver disease.

	LVESD (cm)	LVEDD (cm)	LAD (cm)	EF (%)	SF (%)
CTP class A (*n* = 29)	3 ± 0.6	4.9 ± 0.7	3.4 ± 0.4	67 ± 8.4	36.8 ± 6.9
CTP class B (*n* = 39)	3.4 ± 0.4	5.1 ± 0.5	3.4 ± 0.5	65.9 ± 9.6	34.7 ± 6.3
CTP class C (*n* = 24)	3.3 ± 0.6	5.1 ± 0.7	3.3 ± 0.3	62.3 ± 9.1	33.6 ± 7.2
*t-*test					
CTP class A versus B	*P* = NS	*P* = NS	*P* = NS	*P* = NS	*P* = NS
CTP class A versus C	*P* = NS	*P* = NS	*P* = NS	*P* = NS	*P* = NS
CTP class B versus C	*P* = NS	*P* = NS	*P* = NS	*P* = NS	*P* = NS

CTP: Child-Turcotte-Pugh; LVESD: left ventricular end-systolic diameter; LVEDD: left ventricular end-diastolic diameter; LAD: left atrial diameter; EF: ejection fraction; SF: shortening fraction; NS: nonsignificant. All results are expressed as mean values ± standard deviation.

**Table 2:** Comparative assessment of left ventricular diastolic filling indices with regard to severity of liver disease.

	E-velocity (cm/sec)	A-velocity (cm/sec)	E/A ratio	EDT (msec)
CTP class A (*n* = 29)	56.5 ± 12	50.2 ± 10.2	1.2 ± 0.5	156.7 ± 27.8
CTP class B (*n* = 39)	60.2 ± 15.8	61.3 ± 7.9	1.0 ± 0.3	165.4 ± 13.6
CTP class C (*n* = 24)	62.8 ± 17.2	62.4 ± 15.3	1.1 ± 0.3	196.8 ± 23.5
*t-*test				
CTP class A versus B	*P* = NS	*P* < 0.001	*P* = NS	*P* = NS
CTP class A versus C	*P* = NS	*P* = 0.001	*P* = NS	*P* < 0.0001
CTP class B versus C	*P* = NS	*P* = NS	*P* = NS	*P* < 0.0001

CTP: Child-Turcotte-Pugh; E-velocity: peak early ventricular filling velocity; A-velocity: peak atrial filling velocity; E/A ratio: early/late filling velocity ratio; EDT: E-wave deceleration time; NS: nonsignificant. All results are expressed as mean values ± standard deviation.

**Table 3:** Prevalence of diastolic dysfunction (DD) among the different stages of liver disease.

	CTP class A (*n* = 29)	CTP class B (*n* = 39)	CTP class C (*n* = 24)
DD stage 1	11/29 (37.9%)	15/39 (38.5%)	10/24 (41.6%)*
DD stage 2	3/29 (10.3%)	5/39 (12.8%)	11/24 (45.8%)**

Total	14/29 (48.3%)	20/39 (51.3%)	21/24 (87.5%)***

*P = NS (0.8107) as compared to CTP class A and B; **P = 0.0009 as compared to CTP class A and B; ***P = 0.001 as compared to CTP class A and B.

**Table 4:** Relationship of various parameters with regard to the presence or absence of diastolic dysfunction on univariate analysis.

Variable	Diastolic dysfunction present (*n* = 55)	Diastolic dysfunction absent (*n* = 37)	*P* value
Male/female	33/19	29/12	NS
Older age (>53 years)	46	18	**0.0005**
Ascites (u/s)	40	19	**0.047**
Splenomegaly	38	22	NS
Hepatic encephalopathy	14	8	NS
Child-Pugh class (C versus A/B)			**0.001**
Class A	14	15	
Class B	20	19	
Class C	21	3	
Etiology of liver disease			
Viral	33	26	NS
Alcohol	15	4	NS
Other	7	7	NS
Hemoglobin (g/dL)	9.2 (4.7–18)	9.7 (4.5–17.3)	NS
Total bilirubin (mg/dL)	1.2 (0.4–11.6)	1.1 (0.5–8.7)	NS
Albumin (g/dL)	2.7 (1.5–5)	3 (1.7–5.1)	NS
Prothrombin time (seconds prolonged)	6 (0–24)	5 (0–27)	NS
Platelet count (per *μ*L)	92400 (15200–425000)	98100 (16100–445700)	NS
Use of betablockers	38	17	**0.032**

**Table 5:** Results of multivariate regression analysis with regard to the presence or absence of left ventricular diastolic dysfunction.

Parameter	*P* value	Odds ratio	95% confidence intervals
Age > 53 years	**0.0078**	**4.1996**	**1.4604–12.0765**
Ascites	NS (0.7110)	0.8031	0.2518–2.5616
Child-Pugh class (C versus A/B)	**0.0415**	**4.6036**	**1.0605–19.9843**
Use of betablockers	NS (0.5710)	1.377	0.4553–4.1640

NS: nonsignificant.
